# Case Report: Long-Term Response to Pembrolizumab Combined With Endocrine Therapy in Metastatic Breast Cancer Patients With Hormone Receptor Expression

**DOI:** 10.3389/fimmu.2021.610149

**Published:** 2021-02-22

**Authors:** Dingyong Wu, Shu Tang, Rong Ye, Dongmei Li, Dejian Gu, Rongrong Chen, Huan Zhang, Jianguo Sun, Zhengtang Chen

**Affiliations:** ^1^Institute of Cancer, Xinqiao Hospital, Army Medical University, Chongqing, China; ^2^Geneplus-Beijing, Beijing, China

**Keywords:** breast cancer, immunotherapy, endocrinotherapy, HR positive, T cell receptor repertoire

## Abstract

Breast cancer is one of the most commonly diagnosed malignancies. Although endocrine therapy improves the survival of patients with hormone receptor (HR)-positive breast cancer, the post-endocrine therapy strategy for metastatic breast cancer remains challenging. Herein, we report two patients who benefited from antiestrogen agents combined with an immunotherapy regimen to support the notion that an immunotherapy combination regimen may be a potential treatment for patients with HR-positive metastatic breast cancer post-endocrine therapy. Case 1 involved a patient with relapsed breast cancer with ovarian and brain metastases after endocrine therapy. After undergoing surgery for the ovarian lesions, she received three cycles of chemotherapy. Given that the lesions in the brain did not change, chemotherapy was discontinued. A high T cell receptor (TCR) repertoire (high Shannon index and clonality) was observed in the tumor. Considering the patient's preference and safety, and the efficacy of immunotherapy, she was administered with letrozole combined with pembrolizumab. The patient achieved a partial response, and the progression-free survival (PFS) was more than 21 months. Case 2 involved a patient with breast cancer with multiple bone metastases. After failure of combined radiotherapy and chemotherapy, the patient received tamoxifen combined with pembrolizumab based on the patient's preference and clinical biomarkers of a positive differentiation cluster of eight tumor-infiltrating lymphocytes and a high TCR repertoire (high Shannon index and clonality) in the tumor. The patient's bone pain and biomarkers were relieved after the treatment. The patients completed six cycles of pembrolizumab, and the PFS was more than 21 months. In conclusion, our study confirmed that antiestrogen agents combined with an immunotherapy regimen is a promising treatment for patients with HR-positive metastatic breast cancer.

## Introduction

Breast cancer (BC) is the most commonly diagnosed cancer in women worldwide and is also a leading cause of cancer-related death. BC treatment consists of locoregional treatment (i.e., surgery) and systemic therapy (i.e., endocrine therapy). In patients with hormone receptor (HR)-positive BC, prognosis has substantially improved after the introduction of endocrine therapy (1). Even so, ~20% of patients continue to experience disease recurrence (2). Moreover, endocrine resistance inevitably occurs in estrogen receptor (ER)+ metastatic BC (MBC). Endocrine resistance is commonly driven by ligand-independent ER reactivation (3) which can occur through gain-of-function mutations in ERs; altered interactions of ERs with coactivators/corepressors; or via engagement of compensatory crosstalk among ERs, growth factor receptors, and oncogenic signaling pathways (4). Therefore, treatment options become more complex after endocrine therapy.

Several studies have indicated that endocrine therapy combined with cyclin-dependent kinase 4/6 (CDK4/6) inhibitors (palbociclib, ribociclib, and abemaciclib) significantly improves survival in patients who are resistant to endocrine therapy (5, 6). The median progression-free survival (PFS) is more than 9 months (7). Recently, phosphatidylinositol 3-kinase (PI3K) inhibitors (alpelisib) combined with endocrine therapy have also been shown to be an alternative treatment (8). However, alpelisib was not available in China in 2018.

Recently, preclinical and clinical data have supported the key role of immunotherapy for BC (9). Although <10% of patients with metastatic disease will respond to monotherapy (10), immunotherapy combinations have shown potential benefits in triple-negative BC (TNBC) and human epidermal growth factor receptor 2 (HER2)-positive BC (11, 12); for example, atezolizumab plus nab-paclitaxel in untreated metastatic TNBC showed an objective response rate (ORR) of 56% in the IMpassion130 study (11). These studies have demonstrated the clinical benefits and acceptable safety of immunotherapy for BC, even in heavily pretreated patients with metastatic BC and patients with HR-positive BC.

Here, we propose a new immunotherapy combination regimen for ER+ metastatic BC by presenting two patients who benefited from antiestrogen agents combined with an immunotherapy regimen. Both patients showed benefits lasting longer than 21 months.

## Case Presentation

**Figure 1:**
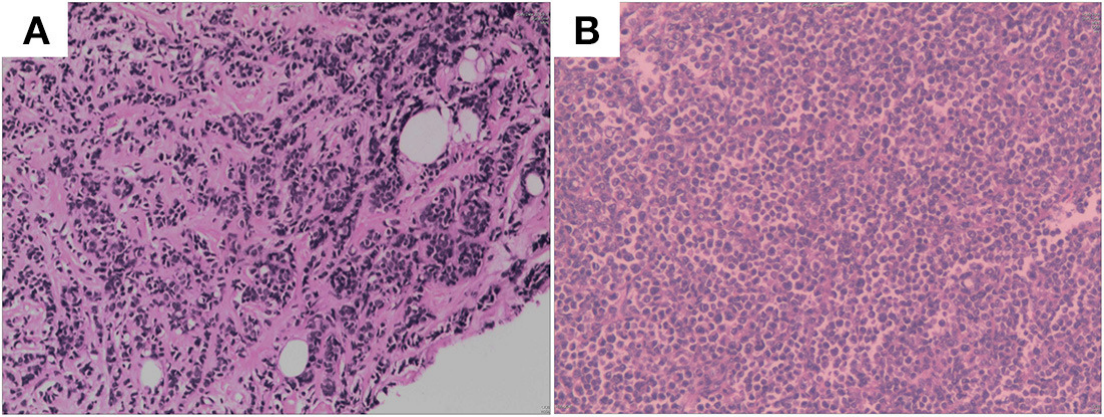
**(A)** Hematoxylin and eosin-stained section of primary lesion. **(B)** Hematoxylin and eosin-stained section of metastasis lesion of ovary.

**Figure 2:**
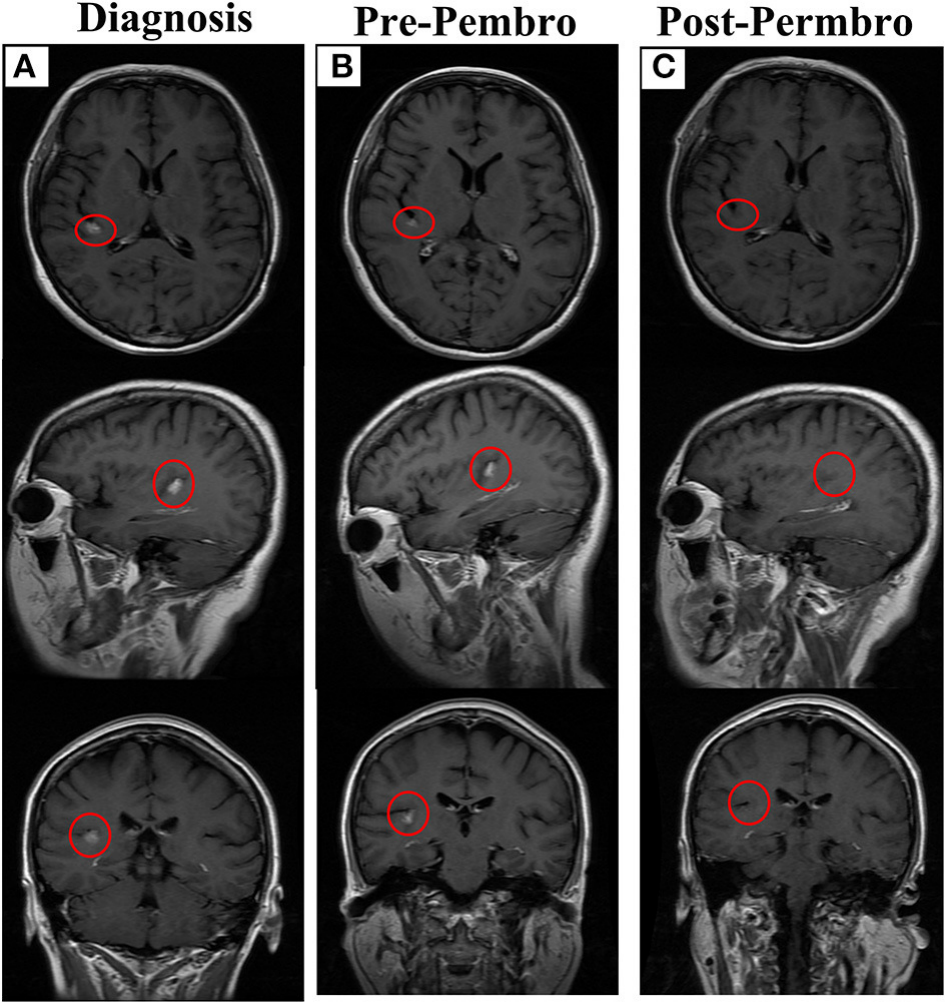
The outcome of letrozole combined with pembrolizumab. **(A)** The brain lesion in diagnosis, lesion has been marked with red circle. **(B)** The brain lesion before treatment with letrozole combined with pembrolizumab. **(C)** Disappearance of brain lesion after treatment. Permbro, pembrolizumab.

Case 1: In March 2018, a 48-year-old woman presented to our hospital with bloody urine. She underwent modified radical mastectomy and received adjuvant chemotherapy and tamoxifen for lobular BC (luminal B subtype) 3 years prior (Figure 1A, Table 1). Color Doppler ultrasonography showed a 10.1 cm × 6.2 cm mass in the left ovary and a 4.3 cm × 3.1 cm mass in the right ovary. Cytoreductive surgery was performed; however, the postoperative pathological diagnosis revealed metastatic lobular BC. Immunohistochemical (IHC) staining showed ER+, progesterone receptor (PR)+, HER2 (1+, fluorescence *in situ* hybridization-negative), and a Ki-67 index of 35% (Figure 1B). A 1.1 cm × 0.6 cm metastatic nodule was found in the right temporal lobe on magnetic resonance imaging (MRI) (Figure 2A). Finally, she was diagnosed with metastatic lobular BC (ovarian and brain). The patient was then treated with three cycles of navelbine and gemcitabine combined with recombinant human endostatin from April to June 2018. Considering the lack of improvement in the brain lesion (Figure 2B), we performed next-generation sequencing (NGS) of 1021 cancer-related genes, an evaluation of the T cell receptor (TCR) repertoire, and IHC analysis of programmed death ligand 1 (PD-L1) (Geneplus-Beijing Ltd., Beijing, China) to identify potentially actionable mutations and biomarkers; the tumor-infiltrating lymphocytes (TILs) were also analyzed using IHC methods. Six somatic mutations, including cyclin D1 (*CCND1*) amplification, were identified (Supplementary Table 1). The results of the TCR repertoire evaluation were 7.47 on the Shannon index of blood and 0.53 for clonality of tissue, both of which were higher than those in 75% of patients with solid tumors in the Geneplus TCR repertoire database (Table 2 and Supplementary Methods); however, PD-L1 (PD-L1 SP263, Ventana) and TILs tested negative (Supplementary Figure 2). Due to difficulties in accessing CDK4/6 inhibitors at that time in China, a combination of CDK4/6 inhibitors and endocrine therapy was not recommended. After comprehensive consideration of the safety and efficacy of immunotherapy, testing results, and patient's preference, a combination of letrozole (2.5 mg, qd) and pembrolizumab (200 mg, q3w) was administered in July 2018. After 3 months of treatment, MRI showed the disappearance of the lesion in the right temporal lobe (Figure 2C). According to the Response Evaluation Criteria in Solid Tumors v1.1, a partial response (PR) was achieved. In October 2018, the patient completed four cycles of pembrolizumab and letrozole. She was then maintained with letrozole. During the course of the treatment, no immune-related adverse events (irAEs) were observed in this patient. The treatment lasted for over 21 months from July 2018, and follow-up is ongoing.

**Table 1:** The clinical characteristic and treatment of patients.

**Patients**	**Date of diagnosis**	**Age at diagnosis**	**Clinical diagnosis**	**Stage**	**Metastases**	**IHC**	**TILs**	**Luminal subtype**	**Treatment initiation time**	**Treatment**	**Duration of treatment**
Case 1	2015-05	45	Breast cancer	N/A	N/A	ER(+), PR(+), HER2 (2+)	N/A	Luminal B	2015-09	Modified radical mastectomy	N/A
									2015-09	TAC^1^ + tamoxifen	30 months
	2018-03	45	Breast cancer	IV	Ovarian and brain	ER(+), PR(+), HER2 (1+), Ki-67(35%)	Negative	Luminal B	2018-04	Cytoreductive surgery	N/A
									2018-04	NG + human endostatin	2 months
									2018-07	Letrozole + pembrolizumab	21 months
Case 2	2018-04	42	Breast cancer	IV	Bbone	ER(+), PR(+), HER2 (–), Ki-67(5%)	Intratumoral:1% CD3+; 1% CD8+ Stromal:1% CD3+; 1% CD8+	Luminal A	2018-05	TAC^2^ + radiotherapy	2 months
									2018-07	Tamoxifen + pembrolizumab	21 months

N/A, not applicable; TILs, tumor-infiltrating lymphocytes;

1 TAC: docetaxel, doxorubicin, cyclophosphamide;

2 *TAC: doxorubicin liposomes, taxol liposomes, cyclophosphamide; NG, navelbine, gemcitabine*.

**Table 2:** The results and range in the Geneplus database of TCR repertoire of the two patients.

**Patients**	**TCR repertoire**	**Sample of detected**	**Results**	**Range of patient**	**Cutoff of data in Geneplus database**		
					**75%**	**50%**	**25%**
Case 1	Shannon index	Blood	**7.47**	**>75%**	7.44	6.42	5.37
	Clonality	Tissue	**0.53**	**>75%**	0.23	0.15	0.11
Case 2	Shannon index	Blood	**6.5**	**>50%**	7.44	6.42	5.37
	Clonality	Tissue	**0.67**	**>75%**	0.23	0.15	0.11

**Figure 3:**
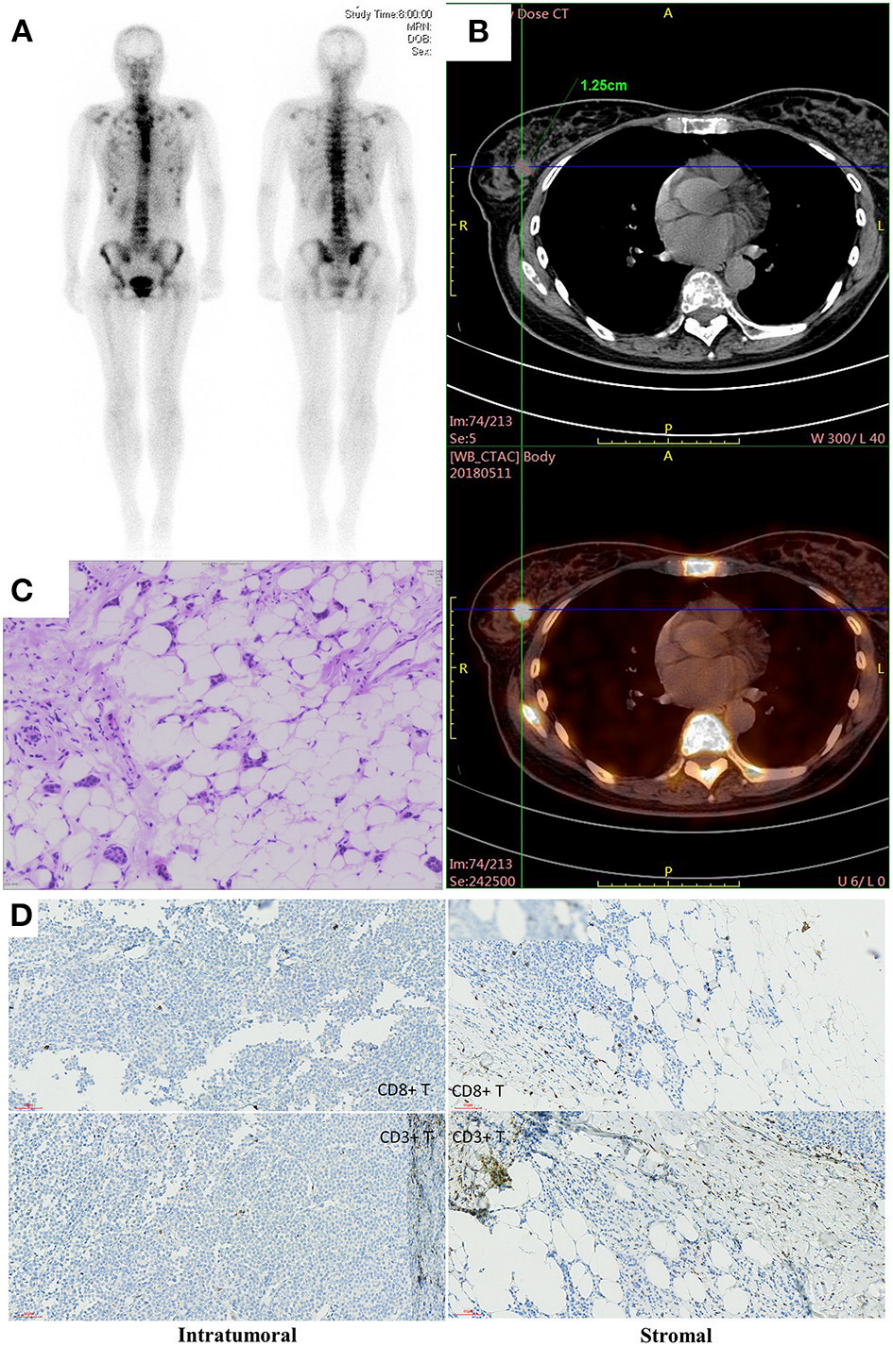
**(A)** The image of whole body bone scans. **(B)** The CT image of breast lesion. **(C)** Hematoxylin and eosin-stained section of breast lesion. **(D)** Immunohistochemistry of intratumornal and stromal CD8 and CD3 T cells.

**Figure 4:**
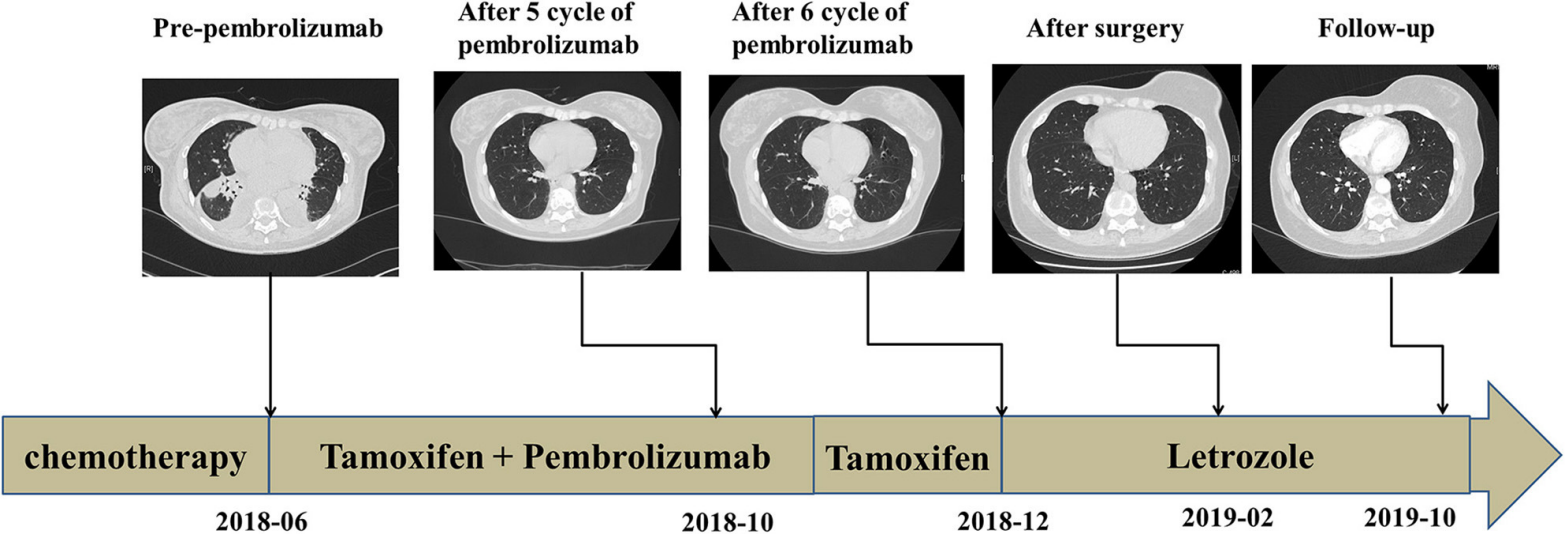
Follow-up imaging of lung lesions at different stages of treatment.

Case 2: A 42-year-old woman presented to our hospital with chest pain. Whole-body bone single-photon emission computed tomography showed multiple lesions with increased radioactivity in the sternum, ribs, centrum, and ospelvicum (Figure 3A). Positron emission tomography/computed tomography (CT) showed a mass in the right breast (Figure 3B). The breast biopsy confirmed adenocarcinoma, and IHC staining showed ER+, PR+, HER2–, and a Ki-67 index of 5% (Figure 3C, Table 1). Therefore, the diagnosis of phase IV, luminal A BC was confirmed. Doxorubicin liposomes, taxol liposomes, and cyclophosphamide were administered as first-line treatment in May 2018. Thoracic radiotherapy was also administered. The patient discontinued chemotherapy due to bone marrow involvement in June 2018. The TCR repertoire and 1,021 cancer-related genes were tested using NGS to identify potentially actionable mutations and biomarkers (Geneplus-Beijing Ltd., Beijing, China), and TILs were also detected using IHC methods. Four somatic mutations including a phosphatidylinositol-4,5-bisphosphate 3-kinase catalytic subunit alpha (*PIK3CA*) p.H1047R were identified (Supplementary Table 1). The results of the TCR repertoire evaluation were 6.5 on the Shannon index of blood and 0.67 for clonality of tissue, which were higher than those in 50 and 75% of patients with solid tumors, respectively (Table 2). Approximately 1% cluster of differentiation (CD)3+ and 1% CD8+ T cells were observed intratumorally and in the stroma, respectively (Figure 3D). Due to the *PIK3CA* mutation, endocrine monotherapy may have had limited efficacy. However, PI3K inhibitors were difficult to obtain in China at that time. Based on these results, the safety and efficacy of immunotherapy, and the patient's preference, tamoxifen (10 mg, bid) combined with pembrolizumab (200 mg, q3w) was administered in July 2018. In November 2018, six cycles of pembrolizumab plus tamoxifen were completed, and she continued with tamoxifen monotherapy thereafter; there were no irAEs other than mild asthenia. The patient's physical status improved significantly. Her Eastern Cooperative Oncology Group performance status improved from 1 to 0, and the bone pain was relieved from 5 to 0 on the numeric rating scale. Follow-up CT showed that the lesions in the bone were stable; however, no efficacy evaluation was established due to the presence of systemic metastasis (Figure 4). In December 2018, letrozole was administered for menopause. Considering that the systemic lesions were well-controlled, the breast lesion was surgically removed. The treatment lasted for more than 21 months from July 2018, and the patient remains in follow-up.

## Discussion

Several studies have confirmed the efficacy of immunotherapy combination therapies for TNBC and HER2-positive BC (11, 12). However, effective combination strategies have not been reported for HR+ BC. The two cases reported here demonstrated promising efficacy of the combination of anti-endocrine and immunotherapy regimens in patients with ER+ BC who had been heavily pretreated with chemotherapy and endocrine therapy.

HR+ BC is considered immunologically “cold” due to low numbers of TILs (13). Additionally, the efficacy of single-agent therapy with pembrolizumab has been evaluated in ER+/HER2-advanced BC; only 20% of ER+ BC cells express the PD-L1 immune checkpoint protein, and single-agent immune checkpoint inhibitors have shown limited efficacy (ORR: 12%, 3/25) in patients with ER+ BC (14). Nevertheless, this study confirmed that immunotherapy is safe in patients with ER+ BC. A recent study confirmed that durvalumab plus bevacizumab therapy is effective for ER+ advanced BC; although no patients achieved complete response or PR, the disease control rate was 37.5% (6/16), and the combined regimen was safe (15). Based on the safety of immunotherapy for ER+ BC and results of studies of an immunotherapy combination regimen for TNBC and HER2-positive BC, our patients were administered immunotherapy plus antiestrogen agents. Moreover, both patients showed a higher Shannon index of blood and clonality of tissue with reference to those in the Geneplus database. The Shannon index represents the diversity of TCR clones in blood, with higher diversity values indicating a more diverse distribution of the receptor sequences. Clonality, which ranges from 0 to 1, is a metric of T cell expansion, with values approaching 1 indicating that few clones are present at high frequencies. All indices are positively correlated with the efficacy of immunotherapy (16–18). Studies on lung cancer and melanoma have indicated

that patients in higher Shannon index or clonality groups showed better efficacy of immunotherapy (16–18). Meanwhile, a recent study showed that a significant proportion of T cell clones and neoantigens are shared between primary lesions and metastases (19), indicating that most lesions may be effectively controlled if immunotherapy is effective. Meanwhile, ERα has been shown to negatively regulate PD-L1 expression (20). Antiestrogen treatment may induce PD-L1 expression and synergize with immunotherapy. Therefore, these findings and studies suggest that immunotherapy plus antiestrogen agents may have been an advantageous treatment option for our two patients with multiline treatment failure and multiple metastases. As expected, both patients showed benefits and had a PFS of more than 20 months following immunotherapy.

In addition, each patient had specific molecular and clinical characteristics. The first patient had luminal B subtype BC and experienced endocrine therapy failure. She benefited from immunotherapy despite negative PD-L1 expression and TILs in the primary tumor biopsy. Considering the heterogeneity of the immune microenvironment between lesions (19), the benefit of the brain lesions from immunotherapy did not conflict with the PD-L1 and TIL results. Furthermore, a recent study demonstrated that high expression of the immune checkpoint components indoleamine-pyrrole 2,3-dioxygenase, lymphocyte-activation gene 3, and programmed death 1 may be induced in luminal B BC, especially after aromatase inhibitor (AI) treatment (21). The study indicated that immune checkpoint inhibitors may be effective for luminal B subtype breast tumors that are being treated with or even resistant to AIs. The second patient had luminal A subtype BC with a *PIK3CA* mutation (p.H1047R). The *PIK3CA* p.H1047R mutation has been shown to be a gain-of-function mutation in several studies and plays an important role in endocrine therapy resistance (22, 23). It has been reported that CD8+ TILs are significantly more abundant in *PIK3CA*-mutated ER-positive BC than *PIK3CA*-wild-type (24). CD8-positive lymphocytes are one of the main components of TILs and are correlated with the efficacy of immunotherapy (25). Meanwhile, CD8+ T cells, although only comprising 1%, were observed in this patient. Sasha et al. reported that only 43% of HR+ tumors have CD8+ T-cell infiltrates (26). The number of CD8+ TILs is a robust predictor of clinical outcomes and treatment response, including immunotherapy in patients with BC (25, 27, 28). The molecular and clinical characteristics of these patients also suggest that they may benefit from immunotherapy regimens. Both of our patients showed a PFS of more than 20 months, which was much longer than the reported median duration of response of approximately 12 months following pembrolizumab monotherapy (14, 19).

Based on these findings, we speculated that a higher TCR repertoire may be related to a better prognosis following immunotherapy in patients with HR+ BC. Our results provide preliminary confirmation of the feasibility and effectiveness of antiestrogen agents combined with an immunotherapy regimen for HR+ BC.

## Conclusion

This represented the first study reporting radiographic partial response following an antiestrogen combined with immunotherapy regimen in breast cancer patients to the best of our knowledge. Our study provides unequivocal clinical evidence for antiestrogen combined with immunotherapy effectiveness in treating ER+ estrogen-resistance breast cancer patients.

## Data Availability Statement

The original contributions presented in the study are included in the article/Supplementary Material, further inquiries can be directed to the corresponding author/s.

## Ethics Statement

Written informed consent was obtained from the individual(s) for the publication of any potentially identifiable images or data included in this article.

## Author Contributions

ZC and JS designed the investigation, and contributed to writing the paper. RY and DL performed the investigation. DG, RC, and HZ provided essential assistance and analyzed data. DW and ST designed and performed the research, supervised the study, analyzed data, and wrote the paper. All authors contributed to the article and approved the submitted version.

## Conflict of Interest

DG, RC, and HZ were employed by company Geneplus-Beijing Ltd. The remaining authors declare that the research was conducted in the absence of any commercial or financial relationships that could be construed as a potential conflict of interest.

## Supplementary Material

Supplementary Figure 1 The distribution of TCR repertoire Shannon index **(A)** and Clonality **(B)** in the Geneplus database of 342 solid tumor patients. The median is indicated by the thick horizontal line and 95% CI is indicated by the thin horizontal line.

Supplementary Figure 2 The IHC results of PD-L1 **(A)** and TILs **(B)** of Case 1.

Supplementary Table 1 Gene mutations detected by NGS of the two patients.
