# Supplementary figures and images for: Case Report: Long-Term Response to Pembrolizumab Combined With Endocrine Therapy in Metastatic Breast Cancer Patients With Hormone Receptor Expression
Source: Front Immunol. 2021 Feb 22;12:610149. doi: 10.3389/fimmu.2021.610149 (PMC7939121; doi:10.3389/fimmu.2021.610149)

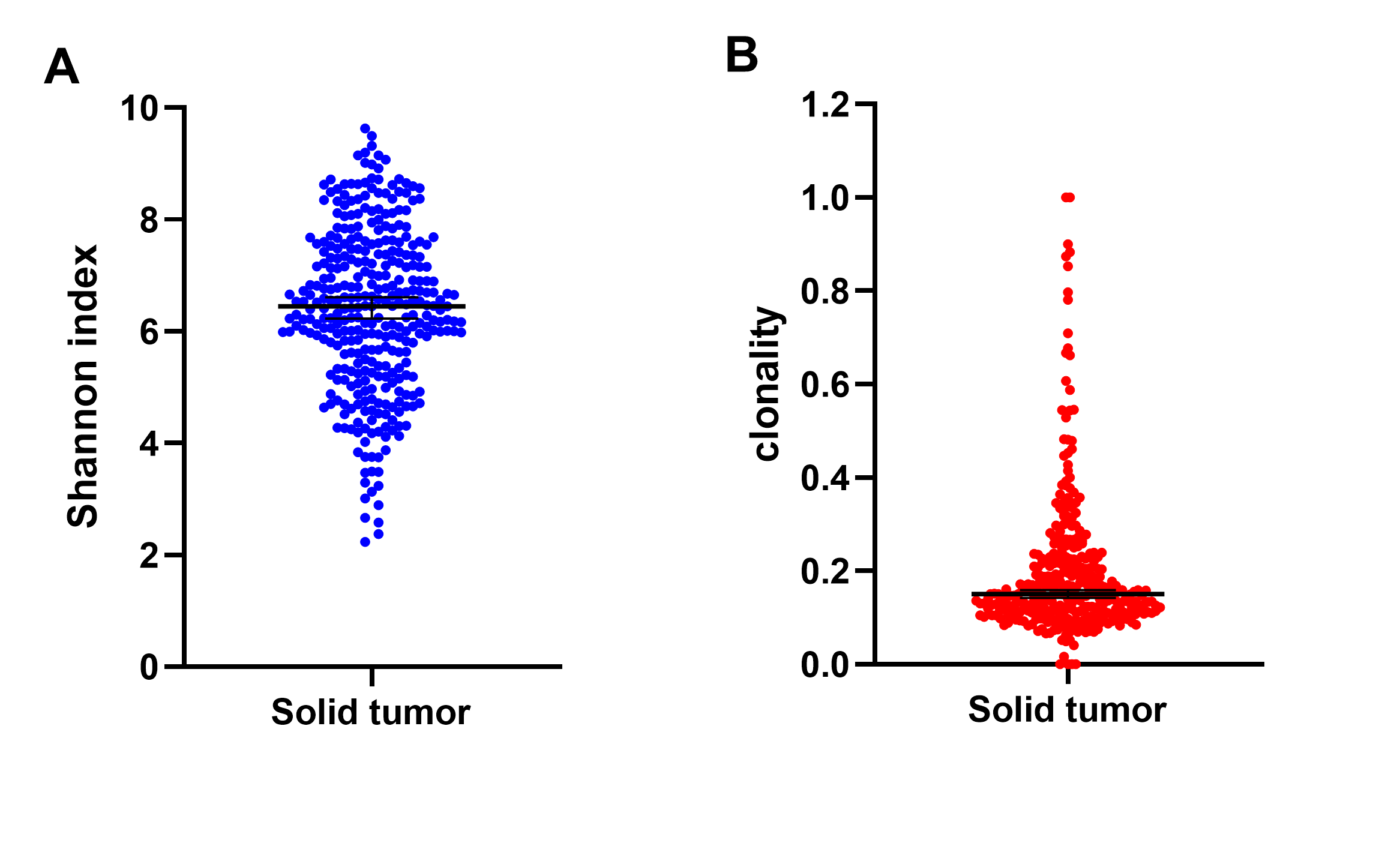

Supplement: Supplementary Figure 1 — The distribution of TCR repertoire Shannon index (A) and Clonality (B) in the Geneplus database of 342 solid tumor patients. The median is indicated by the thick horizontal line and 95% CI is indicated by the thin horizontal line. [file Image_1.tif]

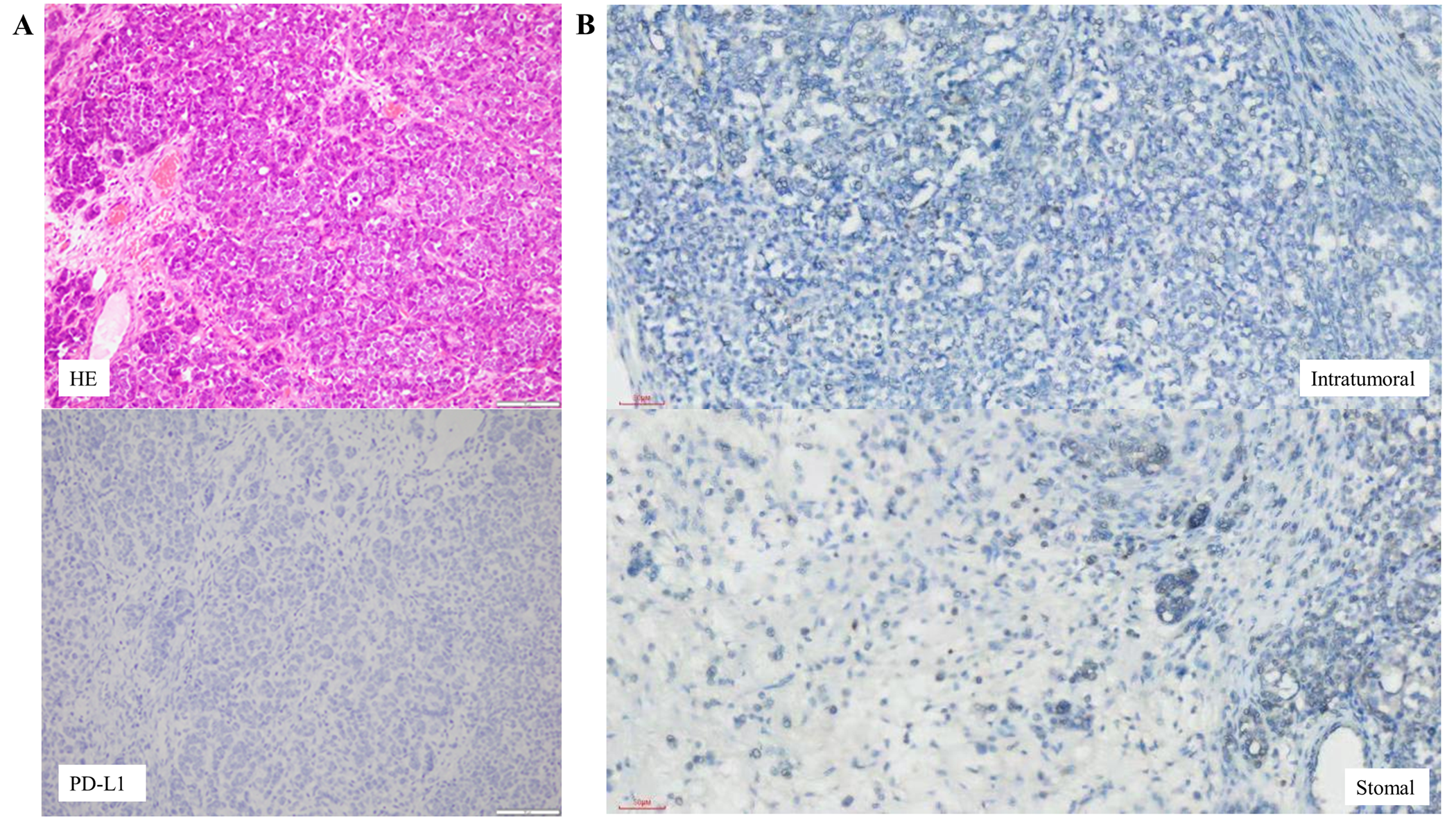

Supplement: Supplementary Figure 2 — The IHC results of PD-L1 (A) and TILs (B) of Case 1. [file Image_2.tif]
