# Supplementary material for: Case Report: Long-Term Response to Pembrolizumab Combined With Endocrine Therapy in Metastatic Breast Cancer Patients With Hormone Receptor Expression
Source: Front Immunol. 2021 Feb 22;12:610149. doi: 10.3389/fimmu.2021.610149 (PMC7939121; doi:10.3389/fimmu.2021.610149)
Supplement: Supplementary Table 1 — Gene mutations detected by NGS of the two patients. [file Table_1.docx]

**Table 1.** Gene mutations detected by NGS of two patients.

| **Case** | **Gene** | **cHGVS** | **pHGVS** | **Exon ID** | **Mutation frequency/copy numbers** |
| --- | --- | --- | --- | --- | --- |
| **case 1** | *CDH1* | c.788C[3>2] | p.Q264Rfs*18 | EX6 | 58.70% |
|  | *MEN1* | c.485_501delTTGGGGCCTGCCAGGCCinsCCA | p.V162Afs*13 | EX3 | 43.50% |
|  | *USP6* | c.2021G>A | p.R674K | EX19 | 35.00% |
|  | *ATRX* | c.1121T>C | p.L374S | EX9 | 2.80% |
|  | *INPP4B* | c.1834T>A | p.Y612N | EX19 | 1.10% |
|  | *CCND1* | amplification | NA | all exon | 7.2 |
| **case 2** | *PIK3CA* | c.3140A>G | p.H1047R | EX21 | 14.30% |
|  | *ERG* | c.277A>T | p.M93L | EX3 | 13.90% |
|  | *ASXL1* | c.1805delA | p.E602Gfs*101 | EX13 | 12.50% |
|  | *FAT1* | c.4592C>T | p.T1531M | EX8 | 1.80% |

ND, not detected; c.HGVS, description of coding DNA (c.) varients by human genome variation society (HGVS); p.HGVS, description of protein (p.) varients by HGVS.

*describe a stop codon.
