# Supplementary material for: Case Report: Long-Term Response to Pembrolizumab Combined With Endocrine Therapy in Metastatic Breast Cancer Patients With Hormone Receptor Expression
Source: Front Immunol. 2021 Feb 22;12:610149. doi: 10.3389/fimmu.2021.610149 (PMC7939121; doi:10.3389/fimmu.2021.610149)
Supplement: Supplementary Table 2 — Supplementary methods. [file Table_2.docx]

**Supplementary Method**

### High-throughput sequencing of T-cell receptor β genes

TCR sequencing and TCR repertoire quantification were performed as previous researches (Liu et al., 2019;Han et al., 2020). TCR repertoire was sequenced using patients’ tissues and peripheral blood. First, the samples of TCR sequencing were prepared used a double barcoded strategy to ensure the accuracy of sequencing in multiplex samples. CDR3 in the TCR β chain (TRB) was inclusively and semiquantitatively amplified by multiplex PCR; the multiplex amplification included both first and second rounds of PCR (PCR1 and (PCR2). The primer sequences have been filed as a part of a Chinese patent (CN105087789A). During the first round of PCR (PCR1), 10 cycles were used to amplify CDR3 sequences using specific primers against each V and J gene. In the second round of PCR, PCR was performed using universal primers. For PCR1, template DNA (1500 ng) was amplified after adding 2× QIAGEN Multiplex PCR Master Mix (25 µl), 5× Q solution (5 µl), the forward primer set pool (1 μl), and the reverse primer set pool (1 μl) to form a reaction system by using a Multiplex PCR Kit (QIAGEN, Germany). Then, PCR was performed with 1 cycle of 95°C for 15 min, 10 cycles of denaturation at 94°C for 30 seconds, and 10 cycles of annealing at 60°C for 90 seconds and extension for 30 seconds at 72°C. After a final extension for 5 min at 72°C, the system was cooled to 4°C. The target fragment of the multiplex PCR products was purified with magnetic beads (Agencourt no. A63882, Beckman, Beverly, MA, USA). All PCR1 products were used as templates for the second step of amplification after adding pooled primers (2 µl), Phusion master mix prepared using a Phusion® High-Fidelity PCR Kit (25 µl; New England Biolabs, America), and nuclease-free water to reach a total volume of 50 µl. The reactions were then transferred to a thermal cycler that carried out the following program: one cycle at 98°C for 1 min; 25 cycles of denaturation at 98°C for 20 seconds, annealing at 65°C for 30 seconds and extension at 72°C for 30 seconds; and a final extension at 72°C for 5 min. The samples were then held at 4°C. Size selection was performed by agarose gel electrophoresis (400 mA/100 V, 2 h), and the targeted fragments (between 200-350 bp) were retrieved and purified by a QIAquick Gel Purification Kit (QIAGEN, Germany). The paired-end sequencing of these samples was carried out with a read length of 151 bp using an Illumina HiSeq 3000 platform.

Raw sequencing data were processed and analyzed as follows. 1) Undesired sequences that did not contain the primers were filtered using Cutadapt https://cutadapt.readthedocs.org/, 2) reads were merged to obtain contigs using Pear (https://cme.h-its.org/exelixis/web/software/pear/doc.html), 3) sequences were aligned to reference TRB V/(D)/J gene sequences (http://www.imgt.org) using MiXCR to determine the TRB V/(D)/J gene segment in each contig, 4) the CDR3 region was identified based on the conserved sequence of the CDR3 region, and 5) CDR3 species were clustered to eliminate sequencing errors according to the base quality and sequence similarity. Finally, 1 million reads were randomly selected to complete normalization.

As previously reported(Han et al., 2020), the Shannon index was calculated as follows, where *ni* is the clonal size of the clonotype (that is, the number of copies of a specific clonotype), *S* is the number of different clonotypes, and *N* is the total number of TCR sequences analyzed:

Shannon index=$-\sum_{i=1}^{S} \frac{ni}{N}ln\frac{ni}{N}.$

T cell clonality was defined as 1 − (Shannon index)/ln(# of productive unique sequences). A maximally diverse population has a clonal score of 0, and a perfectly monoclonal population has a clonality score of 1. T cell richness is a metric of T cell diversity, calculated based on T cell unique rearrangements.

### The composition of Geneplus database and Analysis of TCR repertoire

The database as control in our study was composed with 342 patients, was composed of 12 cancer types, including 144 lung cancer, 26 breast cancer, 23 colon cancer, 23 gastric cancer and so on. The TCR repertoire Shannon index and Clonality of these patients were calculated and performed the distribution as a Geneplus database (Supplementary Figure 1). The upper quartile and the median of the distribution were deemed to be cutoff values greater than 75% and 50% of the population. Therefore, the higher TCR repertoire judged in this study was based on the cutoff of this distribution (Table 2).

## Reference

Han, J., Duan, J., Bai, H., Wang, Y., Wan, R., Wang, X., Chen, S., Tian, Y., Wang, D., Fei, K., Yao, Z., Wang, S., Lu, Z., Wang, Z., and Wang, J. (2020). TCR Repertoire Diversity of Peripheral PD-1(+)CD8(+) T Cells Predicts Clinical Outcomes after Immunotherapy in Patients with Non-Small Cell Lung Cancer. *Cancer Immunol Res* 8**,** 146-154.

Liu, Y.Y., Yang, Q.F., Yang, J.S., Cao, R.B., Liang, J.Y., Liu, Y.T., Zeng, Y.L., Chen, S., Xia, X.F., Zhang, K., and Liu, L. (2019). Characteristics and prognostic significance of profiling the peripheral blood T-cell receptor repertoire in patients with advanced lung cancer. *Int J Cancer* 145**,** 1423-1431.
